# Interventions to Improve Health Among Refugees in the United States: A Systematic Review

**DOI:** 10.1007/s10900-024-01400-2

**Published:** 2024-09-06

**Authors:** Leandra Bitterfeld, Mustafa Ozkaynak, Andrea H. Denton, Cornelius A. Normeshie, Rupa S. Valdez, Noor Sharif, Priscilla A. Caldwell, Fern R. Hauck

**Affiliations:** 1 College of Nursing, University of Colorado, Anschutz Medical Campus, Aurora, CO USA; 2 Primary Children’s Hospital, Salt Lake City, UT USA; 3 Claude Moore Health Sciences Library, University of Virginia, Charlottesville, VA USA; 4 Department of Family Medicine, University of Virginia, Charlottesville, VA USA; 5 Department of Systems Information Engineering, University of Virginia, Charlottesville, VA USA; 6 Department of Public Health Sciences, University of Virginia, Charlottesville, VA USA

**Keywords:** Refugees, Interventions, Healthcare, Community health, Asylees

## Abstract

Refugees arriving to the U.S. experience a high burden of both communicable and non-communicable diseases. There is a potential to improve health outcomes for refugees through well-developed, comprehensive interventions, but the effectiveness of such interventions is poorly understood. The purpose of this review is to identify, characterize and evaluate the effectiveness of patient-level healthcare interventions for U.S. refugee populations. We conducted a systematic review according to the Preferred Reporting Items for Systematic Reviews and Meta-Analyses. Medline via PubMed, Web of Science, Embase, and CINAHL were searched for articles that included a population of refugees of any age, included an intervention aimed at improving health, included an evaluation of the intervention’s outcomes, and were conducted in the U.S. from 2000 to 2022. Thirty-seven studies were included, and we identified three main intervention modalities: healthcare provision/management, resource provision, and education. Interventions targeted general health, infectious disease, women’s health, diet/exercise, health literacy, oral health, diabetes, family health, and substance use. The outcomes measured included knowledge, satisfaction, behavioral outcomes, and physical health markers. This review demonstrates that a few health conditions, namely tuberculosis, have been addressed with large-scale, sustained interventions. Other conditions (general health and women’s health) have been addressed through piecemeal, short-term interventions. The evaluation of interventions often focuses on knowledge or satisfaction rather than health or behavior change outcomes. Future work should focus on the best strategies for developing sustainable interventions that meet the needs of the diverse population of refugees in the U.S.

**Supplementary Information:**

The online version contains supplementary material available at 10.1007/s10900-024-01400-2.

## Introduction

Since the passage of the 1980 Refugee Act, 3.2 million refugees have been admitted to the United States (U.S.) [1]. Internationally, refugees are defined as “people who have fled their countries to escape conflict, violence, or persecution and have sought safety in another country” [2]. The U.S. definition of refugees is “s a person who is unable or unwilling to return to their country of nationality (or country of last habitual residence, if stateless) because of persecution or a well-founded fear of persecution on account of race, religion, nationality, membership in a particular social group, or political opinion” [3]. Refugees have not yet entered the U.S., while asylees are defined as those who meet the definition of a refugee but either currently reside in the U.S. or are seeking admission at a point of entry [3]. Refugees come from across the globe, and the highest number of refugees relocating in the U.S. in the last ten years came from Burma, Iraq, the Democratic Republic of Congo, Bhutan and Somalia [3].

Refugees and asylees (hereafter, “refugees”) arriving to the U.S. experience a high burden of both communicable and non-communicable diseases, including diabetes, hypertension, tuberculosis, and hepatitis B [4, 5]. While refugees experience higher rates of communicable diseases than the host country populations [4], rates of non-communicable diseases are similar between refugees and host populations [5]. Refugees, however, experience an increase in the prevalence of non-communicable diseases, rather than a decrease, as time in the country of arrival increases [6]. In the U.S., one study found that for each additional year post-resettlement, refugees had an estimated 12% increased odds of diabetes mellitus and 7% increased odds of hypertension [6]. The contributing factors to this decline in health status include reductions in healthy food intake and exercise, as well as limited health literacy and healthcare access [6]. Thus, addressing both communicable and non-communicable disease is equally important in this population.

Addressing this burden of disease for refugees can be overwhelming, considering the wide range of health conditions deserving of attention and the heterogeneity of cultures within the group of people cast under the umbrella designation of “refugee.” Additionally, the prevalence of diseases among refugees changes in response to overseas interventions, and the geographic makeup of refugees entering the U.S. each year depending on global events and U.S. policy [7].

There is a potential to improve health outcomes for refugees through systematic interventions; however, the effectiveness of prior interventions is poorly understood. Identifying interventions that *consistently* improve health outcomes across various settings and populations is important given the diverse cultural factors and health practices of refugees, as well as the broad range of settings where refugees are resettled, and care is received. Importantly, since refugee health interventions are often culturally tailored, it is important to understand whether effective interventions among a specific refugee group are can also be effective in different refugee populations. Moreover, disseminating the results of effective interventions is vital as refugees are resettled in numerous towns, cities, and communities across the country whose respective refugee resettlement organizations, medical centers, and other aid organizations may not be able to communicate with one another easily. Without a critical synthesis of findings, efforts to improve refugee health are sporadic and may be poorly informed.

We conducted a systematic review to identify patient-level healthcare interventions for refugee populations within the U.S. to (1) identify prior effective interventions and (2) understand where gaps exist in the development of interventions to improve the delivery of healthcare for refugees. To our knowledge, this is the first review addressing this specific topic. Our review allows for an understanding of existing health interventions for refugee populations and can inform new intervention development and their dissemination and scalability. Based on this information, researchers, resettlement agencies, policymakers, and other relevant stakeholders will be able to develop more effective interventions to improve health care for refugees within the U.S.

## Methods

We conducted a systematic review according to the Preferred Reporting Items for Systematic Reviews and Meta-Analyses (PRISMA) [8] protocol to identify all empirical studies of interventions targeted at improving refugee health outcomes within the United States.

### Eligibility Criteria

Studies were included if they met the following criteria: included a population of refugees of any age, included an intervention aimed at improving health, included an evaluation of the intervention’s outcomes, and were conducted in the U.S. Studies were limited to those conducted in the U.S. since healthcare interventions are influenced by the U.S.’s complex multi-payer healthcare system, as well as federal and state policies, existing integration and support services, and funding allocation which are unique from other countries. Studies were excluded if they did not meet inclusion criteria or if the primary outcome was mental health, as several systematic reviews of interventions targeting mental health outcomes have been previously published [9]. Studies were also excluded if the intervention was not evaluated or there were no outcome data; they only evaluated provider and clinic outcomes, only included financial outcomes (cost-effectiveness, etc.), evaluated outcomes from mixed populations (i.e., refugees and immigrants) that did not include a subgroup analysis of the refugee/asylee population, or were case studies.

### Search Strategy and Data Sources

Four databases were searched on January 14, 2023: Medline via PubMed, Web of Science, Embase, and CINAHL. Search terms included a combination of keywords and database-specific subject headings for concepts around (a) refugees and (b) access and/or social determinants of health. Searches were limited to studies published in 2000 to search date, written in English, and conducted in the U.S. The full search strategies can be found in Supplement 1.

Citations were exported from each database and de-duplicated in EndNote (EndNote 20, Clarivate, Philadelphia, PA). The resulting 4,788 citations were clustered via DoCTER Topic Extraction (https://www.icf-docter.com/) into major themes in the results. Of these, citations in three clusters, mental health, law, and migration, were exported into Covidence (Covidence systematic review software, Veritas Health Innovation, Melbourne, Australia. Available at www.covidence.org.) and pre-screened by a single reviewer (AHD) for exclusion. Citations retained from this screening step were combined with the remaining references for 3,278 total citations, which were again imported into Covidence.

### Study Selection

**Fig. 1:**
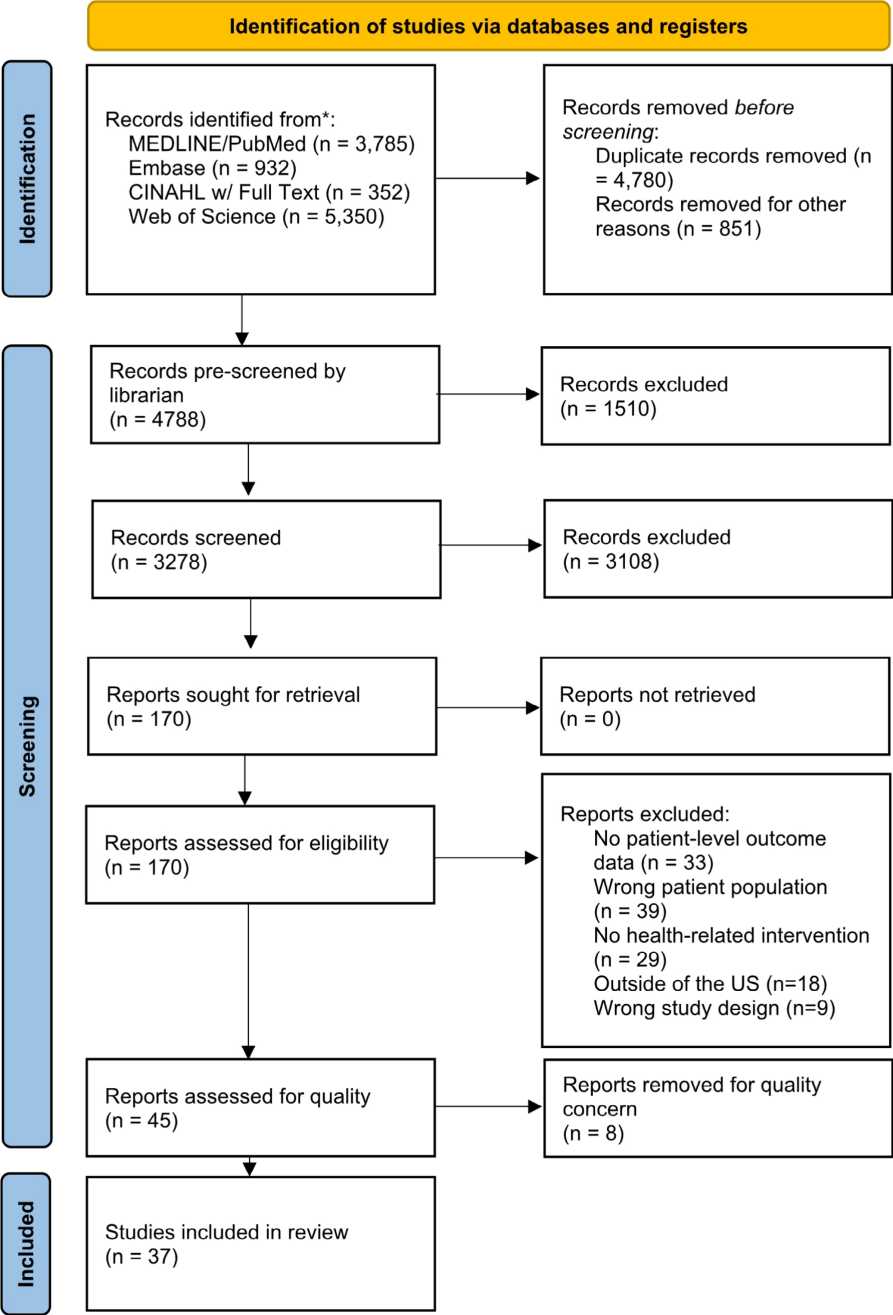
PRISMA review diagram

Titles and abstracts were screened by two authors (LB, PC, CN, MO, and NS) using the above-described inclusion and exclusion criteria. If there was disagreement on whether to include a study, it was either brought to the group for discussion until a consensus was reached or screened by a third independent reviewer. Full-text articles were reviewed by a single author (LB, CN, MO, and NS). This was deemed appropriate since all authors were familiar with the inclusion and exclusion criteria after the lengthy title and abstract review process. If a reviewer was unsure whether to include a full-text article, it was again brought to the group for discussion. The source review PRISMA diagram can be found in Fig. 1.

### Quality Appraisal

After reviewing full texts for inclusion and exclusion, all included articles were appraised for quality using the Mixed Methods Appraisal Tool (MMAT) [10]. The MMAT was chosen (1) to accommodate the wide range of study designs included in the review and (2) because it has demonstrated content validity [11] and adequate reliability [12]. A single reviewer appraised studies for quality, and if the reviewer was uncertain about the appraisal, the study was brought to the group for discussion. Also, all studies that the initial reviewer felt to have a high risk of bias were brought to the group for discussion. Studies deemed to have a high risk of bias were excluded (*n* = 8). In accordance with guidance from the authors of the MMAT [13], studies were awarded one point for a “yes” response to any of the five criteria, which differ based on study design, for an overall score of 0–5. Mixed methods studies were awarded the lowest score of the study components.

### Data Extraction and Analysis

Data was extracted by a single reviewer (LB, MO, CN) using a custom extraction template in Covidence. Information regarding study design, setting, sample characteristics (age, gender, country of origin, time in the U.S.), study duration, intervention target (i.e., healthcare access, infectious disease, oral health, etc.), intervention description, outcomes, and outcome effectiveness were extracted. Due to the substantial heterogeneity of study design, intervention type, and outcomes sought, we conducted a narrative synthesis of study characteristics and findings. The analysis of studies is organized by intervention modality.

## Results

### Study Characteristics

Thirty-seven studies (Table 1) were included in this review, encompassing a broad range of study designs. Included were 11 cohort studies, 9 non-randomized experimental studies, 5 qualitative studies, 4 randomized controlled trials, 3 retrospective studies, 2 quality improvement projects, 2 mixed-methods studies, and 1 cross-sectional study. Most studies (*n* = 30) targeted an adult population; six included children and their parents, and a single study included an older adult population. The sample size also varied widely, from 14 to 3007 participants. Study participants came from 58 different countries, with the most common countries of origin being Bhutan (*n* = 13), Myanmar/Burma (*n* = 11), Somalia (*n* = 9), Iraq (*n* = 9), Afghanistan (*n* = 7), Nepal (*n* = 6), Eritrea (*n* = 6), Democratic Republic of Congo (*n* = 6), Syria (*n* = 6), Sudan (*n* = 5), Ethiopia (*n* = 5), Vietnam (*n* = 4), Burundi (*n* = 4), Cambodia (*n* = 4), and Uganda (*n* = 4).

**Table 1:** Study characteristics

	Author, year	Study Design	Setting	Sample size	Adult/child population	Purpose
1	Alrashdi, Hameed et al., 2021	Randomized controlled trial	Dental clinic	66	Adult/child	Assess the effectiveness of an oral health and behavior intervention program.
2	Alrashdi, Mendez, et al., 2021	Randomized controlled trial	Community	66	Child	Assess a preventive outreach educational intervention on dental caries and oral-health-related quality of life.
3	Berkson et al., 2014	Non-randomized experimental study	Primary care clinic	126	Adult	Describe the impact of the Cambodian Health Promotion Program on health outcomes.
4	Blackstone & Hauck, 2022	Retrospective chart review	Primary care clinic	3007	Adult	Understand telemedicine use patterns among refugees.
5	Carter et al., 2017	Cohort study	Primary care clinic	436	Adult	Understand the effect of a clinical pharmacist on latent tuberculosis therapy completion rates.
6	Culhane-Pera et al., 2005	Non-randomized experimental study	Primary care clinic	39	Adult	Evaluate the influence of group visits on diabetes management in Hmong adults with DM2.
7	Einterz et al., 2018	Non-randomized experimental study	Primary care clinic	384	Adult	To determine the change in early loss to follow-up and time to initiation of latent tuberculosis infection treatment after expansion of a county health department’s refugee screening process.
8	Farokhi et al., 2018	Non-randomized experimental study	Community	151	Adult	Assess the oral health literacy knowledge gained from an oral health literacy intervention.
9	Goldberg et al., 2004	Cohort study	Public Health department	2325	Adult	Understand the effect of cultural case management on TB testing and treatment performance.
10	Goldsmith et al., 2016	Non-randomized experimental study	Refugee resettlement agency	63	Adult	Understand the impact of an educational workshop on refugees understanding of the U.S. pharmacy system.
11	Goodkind, 2005	Cohort study	Community	28	Adult	Assess the effectiveness of a community-based advocacy and learning intervention for Hmong refugees.
12	Higgins, et al., 2019	Retrospective chart review	Primary care clinic	80	Adult	Define the role of the pharmacist working under a collaborative practice agreement in chronic disease state management of refugee patient healthcare.
13	Hoffman et al., 2020	Mixed Methods	Community	50	Adult/child	Assess the feasibility and acceptability of the Refugee Family Cohesion Program parenting intervention.
14	Im & Rosenberg, 2016	Qualitative research	Community	27	Adult	Evaluate the impact of a pilot peer-led community health workshop (CHW) in the Bhutanese refugee community.
15	Im, 2018	Qualitative research	Community	22	Adult	Explore the impact of community-based health workshops, while expanding and re- defining the framework in the context of health promotion efforts for the refugee community in resettlement,
16	Kowatsch-Beyer et al., 2013	Cohort study	Public Health department	224	Adult	Identify the proportion of refugees that were TST-positive, how many attended after referral for medical evaluation, what characteristics influenced follow-up, and whether programmatic changes would increase follow-up rates.
17	Linde et al., 2016	Non-randomized experimental study	Public Health department	4132	Adult	Increase linkage to care for refugees with chronic HBV infection.
18	Maack & Willborn, 2018	Cohort study	Primary care clinic	374, (107 refugees)	Adult	To compare the tobacco use, exposure, and cessation differences between Bhutanese refugee and non-Hispanic White tobacco users in a US federally qualified health center tobacco cessation program.
19	McElrone et al., 2020	Randomized controlled trial	Community	20	Child	Determine the feasibility and acceptability of implementing Pika Pamoja, a culturally adapted childhood obesity prevention program.
20	Michael et al., 2019	Cohort study	Primary care clinic	285	Adult	To assess the impact of the Refugee Health Collaborative on access to coordinated care within patient-centered medical homes.
21	Miner et al., 2017	Cohort study	Community	40	Older adult	Assess the impact of a home health care (HHC) pilot project on meeting the needs of older adult refugee patient.
22	Mosley et al., 2021	Non-randomized experimental study	Community	113	Adult/infant	To evaluate maternal health outcomes, child health outcomes, and breastfeeding intentions among the participants in a refugee birth support program.
23	Ornelas et al., 2018	Cohort study	Community	40	Adult	To develop and evaluate educational videos to promote cervical cancer screening among Karen-Burmese and Nepali-Bhutanese refugees.
24	Percac-Lima et al., 2012	Cohort study	Clinic	95	Adult	Understand the effect of a culturally tailored navigator program on breast cancer screening for Serbo-Croatian speaking women refugees and immigrants.
25	Percac-Lima et al., 2013	Non-randomized experimental study	Primary care clinic	188	Adult	Evaluate whether a Patient Navigation program for refugee women decreases disparities in breast cancer screening.
26	Piwowarczyk & Ona, 2019	Qualitative research	Primary care clinic	14	Adult	To determine the experience of participating in a health promotion. program for refugee and asylum seekers and torture survivors.
27	Prescott et al., 2018	Non-randomized experimental study	Community	282	Adult	To develop a community-based educational workshop to improve medication health literacy in refugees.
28	Rodriguez-Torres et al., 2019	Cross sectional study	Women’s health clinic	126	Adult	To examine the long-term effects of a patient navigation program for mammography screening tailored to refugee women.
29	Scherman et al., 2007	Qualitative research	Community	118	Adult/child	Evaluate a culturally based storytelling as a method to convey farming safety information to Hmong families.
30	Shi, et al., 2019	Cohort study	Primary care clinic; Community	133	Adult	Investigate betel nut usage patterns and the effectiveness of a visually guided educational initiative.
31	Stockbridge et al., 2022	Quality Improvement project	Community	148	Adult	Improve latent tuberculosis infection treatment completion rates
32	Subedi et al., 2015	Cohort study	Primary care clinic	149	Adult	Compare the evaluation and treatment of latent tuberculous infection (LTBI) in refugees seen at member clinics of the Philadelphia Refugee Health Collaborative (PRHC) vs. non-PRHC clinics
33	Vais et al., 2020	Quality Improvement project	Women’s health clinic	78	Adult	Assess the efficacy of a healthcare-directed rideshare application for overcoming attendance barriers at an urban health clinic.
34	Van Zandt et al., 2016	Retrospective program review	Community	144	Adult/infant	Describe outcomes of a birth companion program for vulnerable women.
35	Wagner et al., 2016	Randomized controlled trial	Community	140	Adult	Investigate a community health worker- delivered lifestyle intervention for the prevention of cardiometabolic disease.
36	Wieland et al., 2017	Mixed Methods	Hospital; Primary care clinic	25	Adult	Examine the potential effectiveness of digital storytelling intervention designed through a community-based participatory research (CBPR) approach for immigrants and refugees with type 2 diabetes mellitus (T2DM).
37	Yun et al., 2016	Qualitative research	Community	35	Adult	Identify barriers to care, help-seeking behaviors, and the impact of a community-based patient navigation intervention on patient activation levels.

### Study Quality

Included studies met at least two of the five quality criteria, with most studies meeting four (*n* = 12) or five (*n* = 13) of the quality criteria. Nine studies met three of five quality criteria, and four met two of five. The results of the study quality appraisal can be found in Supplement 2.

### Interventions and Outcomes

A broad range of health conditions were targeted via three main intervention modalities: healthcare provision/management, resource provision, and education. Healthcare provision and management interventions encompassed interventions that provided tailored or modified healthcare. Resource provision interventions consisted of those that provided resources outside of healthcare. Several interventions offered both healthcare and resource provision. Education interventions sought to increase knowledge or awareness of a certain health condition or health behavior. Additionally, two main intervention strategies were identified: cultural/linguistic tailoring of intervention materials and the integration of community members or leaders for intervention delivery. A summary of interventions and their associated outcomes can be found in Table 2.

**Table 2:** Summary of interventions and outcomes

Author, year	Interventions condition/ behavior target:	Intervention modality:	Intervention Description	Linguistic/ cultural tailoring	Community member integration	Outcome	Was the intervention effective?	Results of outcome
Carter et al., 2017	Infectious disease	Healthcare provision/ management; Resource provision	Pharmacy-run tuberculosis treatment clinic with resettlement organization provided transportation if needed.			TB treatment completion	Yes	94.4% completed treatment, compared with 30% prior to clinic.
Einterz et al., 2018	Infectious disease	Healthcare provision or management	A physician-led follow-up visit one month after the initial TB screening.			Mean latent tuberculosis treatment delay (days)	Yes	Pre-intervention, the average time between arrival in the U.S. and ordering of medicines was 116 days (SD = 76.4). Postintervention, the average delay between arrival and prescribing of medicines was 68 days (SD = 43.7, *p* < .001).
						Rate of lost to follow-up	Yes	12.5% (*n* = 28) patients were lost to follow up before the intervention and none were lost to follow-up post intervention.
						Proportion of patients seen by a physician	Yes	48.0% of pre-intervention patients were seen by a physician within 90 days of U.S. arrival, compared with 85.6% of postintervention patients (*P* < .001).
Goldberg et al., 2004	Infectious disease	Healthcare provision or management	Cultural case management: home readings of TB skin test, culturally appropriate TB education, referrals to non-TB health and social services.			Acceptance of treatment (treatment started)	Yes	Overall 88%, range 73–99% between groups
				X	X	Treatment completion	Yes	Average completion rate = 82%, groups ranged 63–94%. Significantly higher than before intervention (37%, *p* < .001)
Kowatsch- Beyer et al., 2013	Infectious disease	Healthcare provision or management; resource provision	Enhanced screening referral program contacted the Columbus Public Health (CPH) TB clinic, sent TB results directly, gave reminder phone calls to refugees and assisted with transportation to follow-up if needed.			Attendance at LTBI follow up	Yes	Attendance at follow-up increased from 53.1–93.5%.
Linde et al., 2016	Infectious disease	Healthcare provision or management	Bilingual care navigators that provide education, make appointments for participants, and arranged transportation.	X		Attendance at one HBV-directed medical appointment after HBV testing	Yes	Linkage to care increased from 64–93% (*p* < .001)
Stockbridge et al., 2022	Infectious disease	Healthcare provision or management	Provision of after-dusk home delivery of a 12-dose latent tuberculosis infection regimen of weekly rifapentine plus isoniazid administered via directly observed preventive therapy	X		Treatment completion rates	Yes	Muslim patients had lower treatment completion rates than non-Muslim patients during Ramadan before program implementation (68.8% vs. 95.4%), whereas rates were comparable postimplementation (95.7% vs. 96.4%; difference-in-difference *P* = .011)
Subedi et al., 2015	Infectious disease	Healthcare provision or management	Treatment of Class B Tuberculosis at refugee health clinics			Prompt and complete treatment of refugees with latent tuberculosis infection (LTBI)	Yes	Refugees receiving care from PRHC clinics were more likely to be screened within 30 days of arrival (OR 4.70, 95%CI 2.12–10.44), attend a follow-up appointment (OR 4.53, 95%CI 1.3-16.27), and complete treatment (OR 9.44, 95%CI 2.39–37.3) than those seen at non-PHRC clinics.
Mosley et al., 2021	Women’s health	Healthcare provision or management; resource provision	A comprehensive, culturally tailored pregnancy support program, where participants receive 8 weeks of no-cost, evidence-based childbirth education classes taught in their language by community liaisons. A volunteer provides transportation to prenatal and postnatal visits, continuous support during labor and childbirth, and social connection.			Labor induction	Yes	Embrace participants had 48% lower odds of labor induction (OR = 0.52, *p* = .025)
						Cesarean delivery	No	Non-significant difference
						Full term gestation	No	Non-significant difference
						Low birth weight	No	Non-significant difference
				X	X	Exclusive breastfeeding	Yes	Relative to the comparison group, the Embrace participants had 65% higher odds of planning to breastfeed exclusively (OR = 1.65, *p* = .028).
Ornelas et al., 2018	Women’s health	Education	An entertainment-education (narrative-based) cervical cancer video intervention, which included four modules: a prologue establishing the main characters and topic; two core segments focusing on logistic barriers to screening and screening procedures; and an epilogue closing the story and reminding viewers of key points.			Cervical Cancer Screening Awareness and Intentions.	Yes	After watching the video, participants were significantly more likely to report having heard of a test for cervical cancer (58–100%, *p* < .001) and a Pap test (45–100%, *p* < .001) and more likely to be screened for ervical cancer (40–80%, *p* < .001).
				X		Cervical Cancer Knowledge.	Yes	Participants had a higher mean composite knowledge scores (5.6 to 9.3, *p* < .001) after viewing the video. Increase in knowledge scores were significant for women in each ethnic group (5.4 to 9.2, *p* < .001 for Karen-Burmese and 5.8 to 9.5, *p* < .001 for Nepali-Bhutanese).
Percac-Lima et al., 2012	Women’s health	Healthcare provision or management; Resource provision	Patient navigator who provided motivational interviewing, scheduling support, education, home visits, transportation assistance, insurance navigation and appointment navigation for breast cancer screening.	X	X	Mammogram status	Yes	Proportion of patients with a mammogram increased from 44–67% (*p* = .001). Of twelve patients who had never had mammogram, five obtained one during the patient navigation program.
Percac-Lima et al., 2013	Women’s health	Healthcare provision or management	Patient navigators (PNs) educated women about breast cancer screening, explored barriers to screening, and tailored interventions individually to help complete screening	X	X	Adjusted mammography screening completion rates	Yes	Prior to implementation of the PN program (2008), adjusted mammography screening rates were significantly lower among refugee women (64.1%, 95% CI: 49-77%) compared with English-speaking (76.5%, 95% CI: 69-83%, *p* = .02) and Spanish-speaking (85.2%, 95% CI: 79-90%, *p* < .001) women. After the implementation of the PN program (2009), screening rates increased among refugee women (77.3%, 95% CI: 64-87%) and were similar to screening rates among English-speaking (76.8%, 95% CI: 70-82%, *p* = .93) and Spanish-speaking (82.8%, 95% CI: 76-88%, *p* = .27) women. Rates of screening remained non-inferior in 2010 and 2011.
Rodriguez- Torres et al., 2019	Women’s health	Healthcare provision or management	A refugee Patient Navigator (PN) program including culturally and linguistically appropriate educational materials about breast cancer screening, and a culture and language matched navigator who worked to remove psychological and logistical barriers to screening.	X	X	Mammography screening completion rate	Yes	Screening rates were higher among refugee women (90.5%) than English-speaking women (81.9%, *p* = .006). Differences in screening rates were non-significant after the program ended
Vais et al., 2020	Women’s health	Resource provision	Women with gynecologic visits reporting transportation difficulties were offered rides through a healthcare-directed rideshare application	X		No-show rates.	Yes	Of 102 eligible visits, 31 reported transportation insecurity and received rides. Those women had a 6% no-show rate, compared to 30% of women denying transportation barriers and 50% amongst unreachable women (*p* < .0001).
Van Zandt et al., 2016	Women’s health	Healthcare provision or management	A student-run birth companion program focused on providing physical, emotional, and information support to women before, during, and after birth.			Maternal Outcomes- C-section, epidural use, pitocin induction, augmentation	No	Newly resettled refugees had a significantly higher likelihood of having a c-section and epidural anesthesia (both *p* < .05) when compared to the nonvulnerable (i.e., nonrefugees, English speakers) but no difference in the rate of pitocin induction or augmentation.
						Newborn Outcomes - low birth weight, breastfeeding	No	Each of the vulnerable subgroups had, on average, lower birth weight newborns (*p* < .01). Breastfeeding attempt was found to be significantly lower in the refugees subgroup (*p* < .05).
Blackstone & Hauck, 2022	General health	Healthcare provision or management	Telemedicine during COVID-19			Telemedicine utlilization	No Baseline Comparison	25% of refugee encounters were telemedicine vs. 39% of non-refugee encounters. Refugees with hypertension and diabetes were more likely to use telemedicine. Non-English speaking refugees were less likely to use telemedicine. Active MyChart users were more likely to use telemedicine. Patients with Medicaid were less likely to use telemedicine.
Goodkind, 2005	General health	Education	An education and advocacy initiative that involved one-on-one “learning circles” and advocacy activities by undergraduate students with the goal of transferring advocacy skills to refugees.			Access to resources- Satisfaction with Resources scale and Difficulty Obtaining Resources scale	Yes	Scores increased from 3.18 at T1 to 4.08 at T3 and to 3.22 at T4, linear beta coefficient = 0.97, *p* < .001
						Quality of life- Satisfaction with Life Areas (SLA) scale	Yes	Scores increased from 3.62 to 3.93 from T1 to T4, linear beta coefficient = 0.53, *p* < .05
				X		Psychological well-being- distress and happiness sub-scales of Rumbaut’s Psychological Well-Being Scale	Yes	Distress scores decreased from 1.92 to 1.66 from T1 to T4, linear beta coefficient=-0.73, *p* < .01. Hapiness increased from 1.57 to 1.76, no growth model results reported.
Michael et al., 2019	General health	Healthcare provision or management	Development of an algorithm to streamline the process by which refugee care is coordinated and a patient centered medical home is established.			Time required to establish care in PCMHs	Yes	37.5% reduction in time to establish a PCMH.
						Provider acknowledgment of refugee status	No	There was an 18.1% decrease in acknowledgment of refugee status from year 2 to year 3 (*p* = .0006).
						# of emergency department (ED) visits.	Yes	21.7% decrease in ED visits between year 1 and year 3 (*p* = .006)
						PCMH receipt of medical records	No	No significant difference
Miner et al., 2017	General health	Healthcare provision or management	Home healthcare program that includes screening for depression and anxiety, chronic disease and medication management education, connection to community resources and access to preventative care services.			Anxiety	Yes	Median score decreased from 1.00 to 0 (*p* < .001)
						Pain	Yes	Median scores decreased from 3 to 1 (*p* < .000)
						Management of ADLs	Yes	Nine item scale scored 0–9 with 0 = fully independent. Scores decreased from 4.33 to 2.07 (*p* < .001)
						Medication management	Yes	Scores ranged from 0–3 with 0 = total independence. Scores decreased from 3 to 1 for both oral (*p* = .011) and injectable (*p* < .001) medications.
Piwowarczyk & Ona, 2019	General health	Education	Seven-week health promotion program at a safety-net clinic addressing bio-psychosocial-spiritual needs through experiential course-work.			Social networks	Yes	Participants shared that the education groups helped foster social connections and friendships.
						Tools/Techniques and tools to maintain health and well-being	Yes	The learning sessions taught participants how to identify tools they can use to improve their health.
						Health maintenance	Yes	The intervention helped the participants maintain health behaviors consistently.
Scherman et al., 2007	General health	Education	Culturally based storytelling to offer education about farming safety to Hmong families.	X		New Knowledge	No	Participants did not answer directly, so new knowledge could not be assessed. Many participants gave reasons for continuing unsafe practices.
Yun et al., 2016	General health	Healthcare provision or management	Patient navigation program: an accessible location, accessible hours (10 h/week, weekdays and weekends, appointments not required), trained patient navigators.			Patient Activation (patient activation measure)	Yes	The proportion of “highly activated” patients increased from 5.7–32.4%.
						PROMIS physical health score	No	No significant change.
					X	Healthcare access	Yes	Fewer patients missed appointments due to language barriers (25.8–8.8%), avoided calling the doctor due to a language barrier (31.3–2.9%) or missed an appointment due to not knowing how to use public transportation (22.6–0%).
Berkson et al., 2014	Diet and/or exercise, general health, health literacy	Education	Health promotion program consisting of 5 small-group classes (∼ 8 people per class).			Self-rated health	Yes	Fewer reports of poor health (20% vs. 7.2%, *p* = .001), no or little energy (39.2% vs. 17.6%, *p* = .000), and moderate-extreme body pain (42.4% vs. 29.6%, *p* = .011).
						Self-reported social functioning	Yes	Fewer people reporting 3 + days out of role (36.8% vs. 26.4%, *p* = .042).
						Self-reported health behaviors	Yes	Fewer people reported exercise < 120 min/week (44% vs. 19.2%, *p* = .000), no exercise at all (12.8% vs. 1.6%, *p* = .001), relaxation less than 60 min/week (61.6% vs. 48%, *p* = .004), or did not relax at all (51.2% vs. 36%, *p* = .002).
						Sleep quality	Yes	Fewer people reported < 4 h of sleep/night (25.6% vs. 10.4%, *p* = .000) and daily nightmares (13.6% vs. 3.3%, *p* = .011).
						Health confidence	Yes	Fewer people reported they were not confident health can improve (21.6% vs. 2.4%, *p* = .000), not confident understanding cause of illness (19.2% vs. 2.4%, *p* = .000), not confident can explain problems to doctor (7.2% vs. 0%, *p* = .002, and not confident doctors can understand you (4% vs. 0%, *p* = .025)
				X	X	Depression	Yes	Depression score >/=1.75 decreased (52.8% vs. 44%, *p* = .034).
Im, 2018	Diet and/or exercise, general health	Education	Eight community health workshops cover basic knowledge and skills in healthy eating and nutrition, multi-faceted impacts of trauma and migration, stress and coping, and healthy living.			Individual health promotion knowledge	Yes	Self-reported increase in healthy eating knowledge and confidence in preparing healthy food.
						Health capital at family level	Yes	People report sharing what they learned with family members.
				X	X	Health capital at community level	Yes	Workshops allowed for community gathering and building of a support network.
Im & Rosenberg, 2016	Diet and/or exercise	Education	A psychoeducation nutrition and healthy eating curriculum that was developed and adapted with the help of trained refugee leaders.			Improvement in health promotion	Yes	Participants reported improvement in health knowledge and competency in access to proper health resources, improved health practice, including change in health behaviors and coping and a positive change in perceived or subjective health.
				X	X	Building social capital	Yes	Participants reported building support systems, building community capacity, participating in the group and the community action, and developing leadership skills
McElrone et al., 2020	Diet and/or exercise	Education	Eight 2-hour sessions cooking curriculum that emphasized cultural values of collectivism and community, addressed food security and dietary acculturation experience.			Youth program outcomes	Yes	Increase in cooking skills (d = 2.38), eating together (d = 0.69), setting healthy goals (d = 0.42), cooking self-efficacy (d = 0.34). Decrease in playing together (d=-0.63)
				X		Adult program outcomes	Yes	Increase in cooking, eating and playing together (d = 3.47), and kitchen proficiency (d = 4.95). Decrease in food security (d=-1.03)
Wagner et al., 2016	Diet and/or exercise	Education	An education program delivered by community health workers to prevent and control cardiometabolic disease through traditional Cambodian concepts rooted in Buddhism.			Self-rated health	Yes	Improved from 3.6(0.9) to 3.1(1.0), *p* < .001 on a 1–5 scale, where 1 = excellent, 5 = poor.
						Medication adherence	Yes	The number of contextual encounters was marginally related to better medication adherence at follow-up (*r* = .18 *p* = .09)
						Health knowledge	Yes	Knowledge of diabetes prevention increased from 2.9(1.0) to 4.0(1.0), *p* < .001. Knowledge of stroke increased from 6.5(1.5) to 8.8(2.0), *p* < .001
						Barriers to care	Yes	Decreased from 2.4(0.9) to 1.9(0.9), *p* < .001
				X	X	Rice consumption	No	Rice consumption did not decrease.
Prescott et al., 2018	Health literacy	Education	One 2.5-hour education workshop taught by pharmacy students with slides translated into 11 most common refugee languages. Topics were: how to get medications and use of a pharmacist, how to take medications, general medication safety (adverse effects, storage, drug interactions, sharing medications), and how to read medication labels. After the topics, student teachers answered individual questions and reviewed home medications.	X		Correct responses to medication knowledge questions	Yes	Average correct response rate was 77.8%, 10 of 18 questions had a correct response rate greater than 80%.
Goldsmith et al., 2016	Health literacy	Education	One 60 min workshop and two hands-on sessions about US pharmacy navigation and education.			Pharmacy knowledge measured by self-report survey.	Yes	Significant increases were seen in awareness that an identification card must be brought to the pharmacy when filling a prescription (*P* = .0003), the number of refills for a medication is noted on the medication label (*P* = .004) and one can ask for a translator in a U.S. pharmacy (*P* = .0023).
			A culturally focused education program featuring 2 guides; “A Healthy Mouth for your Baby” and “Healthy Habits for Happy Smiles”. The main techniques were instruction, demonstration and motivational interviewing. Five sessions and four evaluations.			Attitudes toward oral health (9 questions)	No	No significant change.
Alrashdi, Hameed et al., 2021	Oral health	Education		X		Self-reported oral hygiene behavior	No	No significant change.
Alrashdi, Mendez, et al., 2021	Oral health	Education	Educational program of two one-hour classes using visual materials to discuss: fluoride application, oral hygiene, nutrition, oral health, and dental care access, including preventive measures.			Michigan Oral-Health-Related Quality of Life Scale - Parent Version (MOHRQoL-P)	No	No significant differences between the children’s pre- and post- intervention oral-health-related quality of life (interference: beta =-0.0223, 95% CI =-0.0810- 0.0364, *p* = .4562; function: beta=-0.0166, 95% CI=-0.0915-0.0583, *p* = .6638) after adjusting for socioeconomic status and education level.
				X		Oral health assessed using the WHO Oral Health Assessment Form, DMFT for permanent teeth and dmft for primary teeth	No	No significant difference between control and intervention groups for DMFT/dmft score (intervention Beta= -0.2310, 95% CI (-0.5733-0.1113), *p* = .1859), after adjusting for income and education levels,
Farokhi et al., 2018	Oral health	Education	An oral health literacy empowerment program			Participants oral health literacy scores	Yes	Pre-intervention mean(SD) = 50(15.33) and post-intervention mean(SD) = 83.50(16.62), *p* < .00001
Culhane-Pera et al., 2005	Diabetes	Education	Group visits at a community health center for enhanced diabetes management			Self-reported 24-hour diet recall and exercise	No	The self-reported frequency of purposeful exercise activities did not change pre-post intervention, and the frequency of self-reported routine activities decreased (12.3 to 6.1 times/month, *p* < .01). Neither purposeful nor routine activities increased in duration or intensity pre-post evaluation. There were no pre- and post intervention differences of carbohydrates, rice, noodles, meat, vegetables, or fruit consumption.
						Mental health (Hmong Hopkins Symptom Checklist-25 [HHSC-25])	Yes	Participants experienced a significant improvement in the anxiety subscale (0.86 to 0.50, *p* < .05) and total scores (1.04 to 0.77, *p* < .05), and non-significant improvement in depression subscale.
				X	X	Physiological measures (A1C, BMI, BP, Cholestrol, Triglycerides, LDL, HDL, BUN, Creatinine, Microalbumin/creatinine in urine)	No	There were no significant changes in physiological measures.
Higgins, et al., 2019	Diabetes	Education; Medical/ pharmaceutical treatment	Pharmacist visits in a clinic setting which includes review of medical records, assistance with medication access and affordability of medications prior to patients receiving health insurance, medication counseling, chronic disease state management, and medication education.			A1c values	Yes	The average A1c decreased from 10.1–8.3%, which resulted in a difference of 1.8% (*p* < .001). 9 patients with an initial A1C > 8% reached an A1C < 8% after pharmacist intervention.
						Frequency of pharmacy interventions	Yes	Among 66 refugees, 68 new drugs initiated, 48 drugs discontinued, 63 dose changes made to a current medications (51 doses increased and 12 doses decreased), and 51 visits resulting in extensive education > 30 min
Wieland et al., 2017	Diabetes	Education	A culturally and linguistically tailored video message targeted at improving diabetes self-management.			Confidence and motivation	Yes	96% reported they were more confident after watching the video and 92% reported increased motivation for managing their diabetes
				X		Change in A1C value in intervention participants	No	Among Somali participants, there was a non-significant decrease in A1c (-0.3%, *p* = .36)
Hoffman et al., 2020	Family health	Education	A six-session training program to provide tools and information about parenting in the US to refugee and immigrant parents, to facilitate empowerment and family cohesion.			Family communication	Yes	Scores increased by 0.98 (*p* < .001)
						Family satisfaction	Yes	Scores increased by 0.74 (*p* < .001)
				X	X	Parent self-efficacy	Yes	Scores increased by 0.70 (*p* < .001)
Shi, et al., 2019	Substance use	Education	An educational brochure on the risk of betel nut mastication and oropharyngeal cancer risk.	X	X	Understanding oral cancer and the health consequences of chronic betel nut use	Yes	Recognizing betel nut use as harmful increased from 75–100% (*p* = .011) among those familiar with betel nut and increased from 8–100% (*p* < .0001) among those not familiar with betel nut. Knowledge that betel nut could cause cancer increased from 52.3–87.5% (*p* = .005) among those familiar, and from 4–87.5% (*p* < .0001) among those unfamiliar.
Maack & Willborn, 2018	Substance use	Education	Tobacco cessation program consisting of a 30-minute initial appointment with a pharmacist or registered dietician. Follow-up appointments were scheduled by the patient with one of the tobacco treatment specialists, and there is no limit to the quantity of follow-up visits.			Prevalence of tobacco cessation at 1, 4 and 12-weeks	Yes	Cessation rates were 86.4% at 1 week, 67.8% at 4 weeks and 28.8% at 12 weeks. Rates were higher among refugees than non-Hispanic White rates at all time points.

#### Infectious Disease

Seven studies offered healthcare or resource provision to improve infectious disease health outcomes, six of which targeted tuberculosis [14–19] and one which targeted hepatitis B virus [20] treatment. Interventions included enhanced physician follow-up after treatment initiation [15], refugee-specific TB clinics [14, 19], in-home care delivery [16, 18], culturally tailored education [16, 20], streamlining of the treatment referral process [17], and transportation assistance [14, 17, 20]. All seven studies measured some form of treatment completion, including the rate of loss to follow-up [15], treatment completion [14, 16, 18], and attendance to care [17, 19, 20], and all studies reported a significant improvement in their respective outcomes.

#### Women’s Health

Six studies leveraged healthcare or resource provision [21–26] and one used education [27] to improve women’s health outcomes. Three studies targeted breast cancer screening uptake [21–23], one targeted cervical cancer screening [27], two focused on childbirth interventions [24, 25], and one focused on general women’s health [26]. To improve breast cancer screening (mammography) rates, patient navigator programs were consistently successful at improving screening rates among refugee women [21–23]. The single educational intervention targeting cervical cancer screening knowledge resulted in increased knowledge and awareness, but screening rates were not measured [27].

Interventions for childbirth support had mixed effectiveness. A culturally tailored pregnancy support program that included transportation provision had positive results for some outcomes (labor induction, exclusive breastfeeding intentions) but no difference in others (cesarean delivery, gestational age, low birth weight infants) [24]. A student-run birth companion program found no difference in either maternal or newborn outcomes [25].

Finally, an intervention providing transportation through a clinic-assisted rideshare application to gynecological appointments found participants who used the rideshare had a significantly lower rate of no-shows than those who did not [26].

#### General Health

Four studies implemented healthcare provision interventions to improve general health outcomes. Interventions included a patient-centered medical home (PCMH) [28], a patient-navigator program [29], telehealth during the COVID-19 pandemic [30], and a home healthcare program for chronic disease management [31]. All found improvements in at least some of their outcomes of interest which included a decrease in emergency room visits [28], rates of no-shows and language barriers [29], rates of telemedicine use [30], and medication management [31].

Two quantitative education interventions targeted general health through either a small group health promotion program [32] or learning circles [33] and reported improvements in general health [32], energy levels [32], quality of life [33], and access to resources [33]. Three qualitative studies reported on general health themes after education interventions [34–36]. Two health promotion programs resulted in participants reporting that they could share health knowledge with their community [34] and identify tools for health promotion [35]. The final qualitative study assessed a storytelling intervention for enhancing farming safety and found that evaluating effectiveness was challenging because participants avoided answering questions directly and continued to endorse unsafe practices [36].

#### Diet and/or Exercise

Among the five studies targeting diet and exercise, four integrated community members or leaders [34, 37–39], and one did not [32], in various education interventions. The studies that targeted an increase in education or knowledge all reported positive outcomes [34, 37–39]. The studies that measured behavior outcomes reported mixed results. A health promotion program found improvements in exercise among participants [32], while an education program targeting a decrease in rice consumption found no change after the intervention [39]. Finally, a family cooking intervention found that refugee participants reported an increase in cooking and eating together among both children and parents [38].

#### Health Literacy

The three studies targeting health literacy interventions all reported on knowledge outcomes [32, 40, 41]. Two studies sought to improve pharmacy navigation, and both reported an increase in knowledge and awareness of how to use pharmacy services [40, 41]. Another study sought to increase confidence in understanding one’s illness and discussing concerns with a doctor and found that significantly fewer participants reported no confidence in performing these tasks after a health promotion intervention [32].

#### Oral Health

Three studies provided an oral health education intervention [42–44]. Two studies found no significant change in knowledge [42, 43] or oral health-related quality of life [42]. The remaining study found a significant improvement in oral health literacy scores among participants who received an oral health empowerment intervention [44].

#### Diabetes

Two education interventions targeted diabetes management using different education modalities, and both studies included hemoglobin A1C (A1C) as an outcome of interest. A group visit program at a community health center [45] and a video education intervention [46] both found no decrease in A1C. One diabetes intervention leveraged a pharmacist-run clinic, which resulted in most participating refugees had medication changes made, and the average A1C decreased from 10.1 to 8.3% [47].

#### Family Health

Only one educational intervention targeting family health was identified. This study implemented a training program to provide refugee parents with tools and information about parenting in the U.S., and reported that participants experienced increased family communication, satisfaction, and self-efficacy [48].

#### Substance Use

Two educational interventions targeted substance use; betel nut use [49] and tobacco use [50]. First, educational brochure administration regarding betel nut use resulted in more refugees endorsing betel nut as harmful and cancer-causing, but the study did not report on betel nut use behaviors [49]. A tobacco cessation program resulted in refugee cessation rates that were higher than rates among non-Hispanic White participants in the program at all time points [50].

## Discussion

This review identifies interventional studies targeting health outcomes among refugees in the U.S. While the group of studies is relatively small, the types of interventions and reported outcomes are remarkably varied. Interventions fell into two broad categories, education and healthcare or resource provision. Four of the 37 studies reported that the interventions were ineffective, while five reported partial effectiveness, meaning the intervention improved some of the outcomes but not others. In one study, there was no comparison group to measure effectiveness. Interventions were effective in the remaining 27 studies for all reported outcomes.

Interventions fell into three broad categories: healthcare provision/management, resource provision, and education, with quite a bit of diversity within those categories. Within education, for example, offerings included a written handout [49], storytelling [36], and experiential coursework [35], among others. The interventions around resource provision and healthcare provision/management were also diverse but had some shared elements. These included transportation [14, 20, 24, 26], care at home [16, 31] and care coordination or navigation [15, 17, 20–23, 28, 29].

Considering the wide range of health problems that refugee community members may encounter, it can be difficult to select and design interventions that are a priority for the population of interest. For this reason, community members should be involved early in the process of selecting the health problem of greatest importance and designing the accompanying intervention. Community members provide meaningful insights into local health issues and their involvement ensures that research questions are relevant and sensitive to the community’s needs and priorities. While several studies in this review involved community members in the implementation of their intervention, only a few [26, 29, 36, 44, 48] describe if and how community members were involved in the selection and design of the intervention. Future research should make every effort to include members of it’s target population in every stage of study design from conception to execution. This ensures that interventions are first and foremost meaningful to the target population. Additionally, this approach is likely to enhance recruitment and retention efforts, and increase effectiveness of the intervention.

Among this diverse group of interventions, an extensive range of outcomes were reported, which makes synthesizing them infeasible. Most reported outcomes were process measures such as attendance rate, knowledge, or self-reported health outcomes instead of tangible health or behavior outcomes. For example, Shi et al. offered an educational brochure about betel nut use, and while they report that more people recognized betel nuts as cancer-causing, this study provides no insight into whether betel nut use patterns changed [49]. Conversely, Higgins et al. reported on A1C values before and after a diabetes pharmacy intervention [47], which allows for a tangible understanding of short-term health outcomes, though the long-term sustainability of this change is unclear. Although the reasons for not reporting health or behavior outcomes were often not explained in the reports papers, potential reasons could be methodological challenges (i.e., measuring is impractical or infeasible) or those health / behavioral outcomes could not be observed within the study period. To achieve more robust and meaningful outcome data in relation to interventions, researchers can employ design strategies such as extending study periods, integrating standard-of-care clinical data, or utilizing portable technology, such as wearable sensors or smart home assistants that measure the behaviors of interest. These strategies may allow for the collection of outcomes that reflect participants’ health status and may also help assess the sustainability of interventions through long-term, longitudinal data collection.

Along with a wide range of study outcomes, studies reported using various measurement tools to capture outcomes. This was needed in part because refugees are a highly heterogeneous group of people who speak a wide range of languages and possess different levels of literacy and numeracy. While all studies attempted to meet the language and literacy needs of their population, this was not always possible. For example, Goldsmith et al. did not have formal interpreters available for their pharmacy education intervention, so most participants used “peer interpreters” [40]. These interpreters offered over-the-shoulder support during survey administration, presenting a serious risk of bias in their responses. Prescott et al. also administered a paper survey to a group with varying levels of literacy where some people completed it independently, and others used an interpreter to read answers to participants, potentially biasing the results [41].

For studies that did use translated measures, the quality of the translation varied. The COnsensus-based Standards for the selection of health Measurement INstruments (COSMIN) offer a rigorous checklist for assessing the quality of a measure, including the translation methodology [51]. Key steps include forward translation by native speakers of the target language with knowledge of the disease state and backward translation by native speakers of the original language who are naive to the disease state. While most studies described their translation process, almost none described it in adequate detail to assess if these conditions were met, and no studies cited the COSMIN checklist. It is important to consider that measures validated in English must also undergo validation and reliability testing in their translated language and target population before they can be regarded as valid and reliable. Encouragingly, some studies acknowledged their lack of validity testing due to understandable feasibility limitations [48] and recognized it as an important area of future research.

It is challenging to evaluate which components contributed to the outcomes for multi-component interventions, such as those that both modified healthcare provision and offered transportation assistance. In resource-limited settings, where offering several interventions may not be feasible, it is essential to understand which components of an intervention are most impactful. Again, while some studies acknowledge their inability to disaggregate the results by intervention modality [32], no studies have implemented a dismantling study. This type of work is important for dissemination and identifying opportunities to refine further for more effective interventions.

The included studies heavily focus on health care for adults, while children’s health was largely ignored. Only one study involved children [38], and one focused on parents [48]. Refugee children face unique challenges in terms of health conditions, healthcare utilization, and health management at home. In comparison to the general child population, they face higher rates of anemia, elevated lead blood levels, growth abnormalities, and poor oral health [52–58]. Considering that approximately a third of refugees entering the U.S. are children under 14 years [59], the work regarding refugee children is disproportionately low and deserves more attention.

Most healthcare interventions identified in this review were delivered in person and few leveraged digital technologies. Only one study [30] leveraged a remote intervention in the form of telemedicine during the COVID-19 pandemic. Digital health interventions can potentially support further engagement in patients’ health management and shared decision-making. Several systematic reviews find that interventions leveraging remote health monitoring and personnel support effectively improve disease management, such as hypertension management [60] and heart failure [61], in underserved populations. Remote monitoring, treatment, and support may be worth exploring among refugee populations, and must consider access to technology, especially smartphones, the technological literacy and the language needs of the target population. Also, several studies included in this review leveraged community members for care delivery, which was recognized as valuable to participants in both qualitative and quantitative studies. Digital health interventions may not be able to provide the same level of relatedness and effectiveness, which is an additional consideration for refugee populations.

## Conclusion

Refugees in the U.S. face a broad range of health challenges and are forced to navigate a complex, costly healthcare system to receive care. This review demonstrates that while some health conditions, namely tuberculosis, have been addressed with large-scale, sustained interventions, other conditions, namely general health and women’s health, have been addressed through piecemeal, short-term interventions. The evaluation of interventions often focuses on knowledge or satisfaction rather than health or behavior change outcomes. Further work is needed to understand the best strategies for developing and maintaining sustainable interventions that meet the needs of the diverse population of refugees entering the U.S. each year. The success of these interventions must be rigorously measured to capture outcomes most directly associated with improved health.

## Electronic Supplementary Material

Below is the link to the electronic supplementary material.

Supplementary Material 1

Supplementary Material 2

## Data Availability

Not applicable, no original data was generated for this work.
