# Supplementary material for: Interventions to Improve Health Among Refugees in the United States: A Systematic Review
Source: J Community Health. 2024 Sep 6;50(1):130–51. doi: 10.1007/s10900-024-01400-2 (PMC11805879; doi:10.1007/s10900-024-01400-2)
Supplement: Supplementary file 1 — Supplementary Material 1 [file 10900_2024_1400_MOESM1_ESM.docx]

*Supplement 1 – Search Strategy*

Searches were conducted on 11/14/2022

**Medline via PubMed**

(((refugee*[tiab] OR "asylum seeker"[tiab] OR "asylum seekers"[tiab] OR asylee*[tiab] OR "refugees"[MeSH Terms]) NOT ((africa[mesh] OR asia[mesh] OR oceania[mesh] OR europe[mesh] OR "south america"[mesh] OR "central america"[mesh] OR canada[mesh]) NOT ("united states"[mesh]))) NOT ("comment"[Publication Type] OR "editorial"[Publication Type] OR "letter"[Publication Type] OR "meta analysis"[Publication Type] OR "review"[Publication Type] OR "systematic review"[Publication Type])) AND ((access*[tiab] OR accept*[tiab] OR attitude*[tiab] OR aware*[tiab] OR barrier*[tiab] OR boundar*[tiab] OR challenge*[tiab] OR deliver*[tiab] OR disadvantage*[tiab] OR dissatisf*[tiab] OR disparit*[tiab] OR enable*[tiab] OR "gap"[tiab] OR gaps[tiab] OR engage[tiab] OR experience*[tiab] OR facilitat*[tiab] OR "help seeking"[tiab] OR helpseeking[tiab] OR inequality[tiab] OR inequalities[tiab] OR equity[tiab] OR inequity[tiab] OR inequities[tiab] OR knowledge[tiab] OR motivat*[tiab] OR obstacle*[tiab] OR perceiv*[tiab] OR perception*[tiab] OR perspective*[tiab] OR promotion[tiab] OR satisf*[tiab] OR trust[tiab] OR understand*[tiab] OR uptake[tiab] OR usage[tiab] OR unmet[tiab] OR utiliz*[tiab] OR "Delivery of Health Care"[MeSH] OR "Patient Acceptance of Health Care"[MeSH] OR "Social Determinants of Health"[MeSH] OR "Health Status Disparities"[MeSH] OR "electronic access"[tiab] OR "medical record"[tiab] OR "electronic health"[tiab] OR "Medically Uninsured"[MeSH] OR "insurance"[MeSH] OR insurance[tiab] OR insured[tiab] OR uninsur*[tiab] OR underinsur*[tiab] OR "Communication Barriers"[MeSH] OR "Translating"[MeSH] OR "Health Education"[MeSH] OR interpret*[tiab] OR "Professional-Patient Relations"[MeSH] OR education[tiab] OR communication[tiab] OR literacy[tiab] OR "social isolation"[MeSH] OR "social marginalization"[MeSH] OR "social environment"[MeSH] OR "social conditions"[MeSH] OR determinant*[tiab] OR "sociodemographic"[tiab] OR "discrimination"[tiab] OR "caregiv*"[tiab] OR "caregivers"[MeSH] OR "Caregiver burden"[MeSH] OR incarcerat*[tiab] OR Socioeconomic[tiab] OR "socioeconomic factors"[MeSH] OR "Cost of Illness"[MeSH] OR income[tiab] OR employment[tiab] OR workplace[tiab] OR "work-related"[tiab] OR unemploy*[tiab] OR impoverished*[tiab] OR poverty*[tiab] OR cost[tiab] OR costs[tiab] OR neighborhood*[tiab] OR liveable[tiab] OR livable[tiab] OR "environment design"[MeSH] OR "built environment"[tiab] OR "Residence Characteristics"[MeSH] OR housing[tiab] OR foreclosure[tiab] OR eviction[tiab] OR mortgage[tiab] OR "physical activity"[tiab] OR Exercise[MeSH] OR exercise*[tiab] OR "sedentary behavior"[MeSH] OR walkability[tiab] OR walkable[tiab] OR transportation[tiab] OR food[tiab] OR crime[tiab] OR violence[tiab] OR "Treatment Adherence and Compliance"[mesh] OR "non-compliance"[tiab] OR noncompliance[tiab] OR compliance[tiab] OR "non-adherence"[tiab] OR nonadherence[tiab] OR adherence[tiab] OR refusal[tiab] OR "no show"[tiab] OR "non-attendance"[tiab] OR "mobile health"[tiab] OR mhealth[tiab] OR m-health[tiab] OR ehealth[tiab] OR e-health[tiab] OR app[tiab] OR apps[tiab] OR application[tiab] OR applications[tiab] OR software[tiab] OR "digital health"[tiab] OR "patient portal"[tiab] OR "patient portals"[tiab] OR "Mobile Applications"[mesh] OR "Wearable Electronic Devices"[mesh] OR "Patient Portals"[mesh] OR Telemedicine[mesh] OR telehealth[tiab] OR telemedicine[tiab] OR "remote monitoring"[tiab] OR "tele-medicine"[tiab] OR "telehealth"[tiab] OR tele‐health[tiab] OR telecare[tiab] OR "tele-care"[tiab] OR "tele‐homecare"[tiab] OR telehomecare[tiab] OR telemanag*[tiab] OR tele‐manag*[tiab] OR telemonitor*[tiab] OR tele‐monitor*[tiab] OR teleconsultation*[tiab] OR tele-consultation*[tiab] OR telenurs*[tiab] OR tele‐nurs*[tiab] OR "remote consultation"[tiab] OR "virtual clinic"[tiab] OR "virtual clinics"[tiab] OR "virtual visit"[tiab] OR "virtual visits"[tiab] OR "virtual care"[tiab] OR "video consult"[tiab] OR videoconsult*[tiab]))

**Embase (Elsevier)**

(refugee*:ti,ab,kw OR 'asylum seeker':ti,ab,kw OR 'asylum seekers':ti,ab,kw OR asylee*:ti,ab,kw OR 'refugee') AND (access*:ti,ab,kw OR accept*:ti,ab,kw OR attitude*:ti,ab,kw OR aware*:ti,ab,kw OR barrier*:ti,ab,kw OR boundar*:ti,ab,kw OR challenge*:ti,ab,kw OR deliver*:ti,ab,kw OR disadvantage*:ti,ab,kw OR dissatisf*:ti,ab,kw OR disparit*:ti,ab,kw OR enable*:ti,ab,kw OR "gap":ti,ab,kw OR gaps:ti,ab,kw OR engage:ti,ab,kw OR experience*:ti,ab,kw OR facilitat*:ti,ab,kw OR "help seeking":ti,ab,kw OR helpseeking:ti,ab,kw OR inequality:ti,ab,kw OR inequalities:ti,ab,kw OR equity:ti,ab,kw OR inequity:ti,ab,kw OR inequities:ti,ab,kw OR knowledge:ti,ab,kw OR motivat*:ti,ab,kw OR obstacle*:ti,ab,kw OR perceiv*:ti,ab,kw OR perception*:ti,ab,kw OR perspective*:ti,ab,kw OR promotion:ti,ab,kw OR satisf*:ti,ab,kw OR trust:ti,ab,kw OR understand*:ti,ab,kw OR uptake:ti,ab,kw OR usage:ti,ab,kw OR unmet:ti,ab,kw OR utiliz*:ti,ab,kw OR "electronic access":ti,ab,kw OR "medical record":ti,ab,kw OR "electronic health":ti,ab,kw OR insurance:ti,ab,kw OR insured:ti,ab,kw OR uninsur*:ti,ab,kw OR underinsur*:ti,ab,kw OR interpret*:ti,ab,kw OR education:ti,ab,kw OR communication:ti,ab,kw OR literacy:ti,ab,kw OR determinant*:ti,ab,kw OR "sociodemographic":ti,ab,kw OR discrimination:ti,ab,kw OR caregiv*:ti,ab,kw OR incarcerat*:ti,ab,kw OR Socioeconomic:ti,ab,kw OR income:ti,ab,kw OR employment:ti,ab,kw OR workplace:ti,ab,kw OR 'work-related':ti,ab,kw OR unemploy*:ti,ab,kw OR impoverished*:ti,ab,kw OR poverty*:ti,ab,kw OR cost:ti,ab,kw OR costs:ti,ab,kw OR neighborhood*:ti,ab,kw OR liveable:ti,ab,kw OR livable:ti,ab,kw OR 'built environment':ti,ab,kw OR housing:ti,ab,kw OR foreclosure:ti,ab,kw OR eviction:ti,ab,kw OR mortgage:ti,ab,kw OR "physical activity":ti,ab,kw OR exercise*:ti,ab,kw OR walkability:ti,ab,kw OR walkable:ti,ab,kw OR transportation:ti,ab,kw OR food:ti,ab,kw OR crime:ti,ab,kw OR violence:ti,ab,kw OR 'non-compliance':ti,ab,kw OR noncompliance:ti,ab,kw OR compliance:ti,ab,kw OR 'non-adherence':ti,ab,kw OR nonadherence:ti,ab,kw OR adherence:ti,ab,kw OR refusal:ti,ab,kw OR 'no show':ti,ab,kw OR 'non-attendance':ti,ab,kw OR 'mobile health':ti,ab,kw OR mhealth:ti,ab,kw OR m-health:ti,ab,kw OR ehealth:ti,ab,kw OR e-health:ti,ab,kw OR app:ti,ab,kw OR apps:ti,ab,kw OR application:ti,ab,kw OR applications:ti,ab,kw OR software:ti,ab,kw OR 'digital health':ti,ab,kw OR 'patient portal':ti,ab,kw OR 'patient portals':ti,ab,kw OR telehealth:ti,ab,kw OR telemedicine:ti,ab,kw OR "remote monitoring":ti,ab,kw OR "tele-medicine":ti,ab,kw OR "telehealth":ti,ab,kw OR tele‐health:ti,ab,kw OR telecare:ti,ab,kw OR "tele-care":ti,ab,kw OR "tele‐homecare":ti,ab,kw OR telehomecare:ti,ab,kw OR telemanag*:ti,ab,kw OR tele‐manag*:ti,ab,kw OR telemonitor*:ti,ab,kw OR tele‐monitor*:ti,ab,kw OR teleconsultation*:ti,ab,kw OR tele-consultation*:ti,ab,kw OR telenurs*:ti,ab,kw OR tele‐nurs*:ti,ab,kw OR 'remote consultation':ti,ab,kw OR 'virtual clinic':ti,ab,kw OR 'virtual clinics':ti,ab,kw OR 'virtual visit':ti,ab,kw OR 'virtual visits':ti,ab,kw OR 'virtual care':ti,ab,kw OR 'video consult':ti,ab,kw OR videoconsult*:ti,ab,kw OR 'interpersonal communication'/exp OR 'social inequity'/exp OR 'patient attitude'/exp OR 'cultural competence'/exp OR 'ambulatory care'/exp OR 'community care'/exp OR 'health care access'/exp OR 'primary health care'/exp OR 'telehealth'/exp OR 'transcultural care'/exp OR 'health education'/exp OR 'social determinants of health'/exp OR 'medically uninsured'/exp OR 'socioeconomic vulnerabilty'/exp OR 'caregiver'/exp OR 'cost of illness'/exp OR 'environmental planning'/exp OR 'housing instability'/exp OR 'physical activity'/exp OR 'telemedicine'/exp OR 'mobile health application'/exp)

**Web of Science Core Collection**

TS=("refugee*" OR "asylee*" OR "asylum seeker" OR "asylum seekers") AND

(TS="health" OR TS="healthcare" OR TS="health care" OR TS="health service*" OR TS="well being" OR TS="wellness" OR TS="preventive" OR TS="prevention" OR TS=“health maintenance” OR TS="screening" OR TS="outpatient*" OR TS="out patient*" OR TS="general practi*" OR TS="primary care" OR TS="ambulatory care" OR TS=emergency OR TS="clinic" OR TS="clinics" OR TS="hospital*" OR TS="service prov*" OR TS="care prov*" OR TS="acute" OR TS="chronic*" OR TS="adherence" OR TS="health personnel" OR TS="nurse" OR TS="nurses" OR TS="doctor*" OR TS="physician*" OR TS="general practitioner*" OR TS="specialist*" OR TS="surgeon*" OR TS="rehabilitat*" OR TS="occupational therap*" OR TS="physical therap*")

(TS=”Dental” OR TS=”dentist*” OR TS=”caries” OR TS=”periodontal” OR TS=”oral health” OR TI="medication*" OR TI="medicine*" OR TI="pharmac*" OR TI="drug "OR TI="drugs" OR TI="prescrip*" OR TS="medication compliance" OR TS="medication non compliance" OR TS="medication noncompliance" OR TS="medication adherence" OR TS="medication nonadherence" OR TS="pharmacist*" OR TS="pharmacy" OR TS="pharmacies")

(TS=“cardiovascular“ OR TS=“heart“ OR TS=“coronary“ OR TS=“cardiac“ OR TS=“hypertension“ OR TS=“hypertensive“ OR TS=“rheumatic“ OR TS=“atherosclerosis“ OR TS=“arteriosclerosis“ OR TS=“stroke“ OR TS=“hemorrhage“ OR TS=“vascular“ OR TS=“gastrointestinal“ OR TS=“digestive“ OR TS=“Gastroesophageal reflux disease” OR TS=“GERD“ OR TS=“bowel“ OR TS=“stomach“ OR TS=“colon“ OR TS=“intestin*“ OR TS=“colorectal“ OR TS=“constipat*“ OR TS=“gastritis“ OR TS=”peptic ulcer” OR TS=“liver“ OR TS=“hepatitis“ OR TS=“cirrhosis“ OR TS=“pancreas“ OR TS=“pancreatitis“ OR TS=“gallbladder“ OR TS=“fatty liver” OR TS=“Thyroid“ OR TS=“hyperthyroid*” OR TS=“hypothyroid“ OR TS=“diabet*“ OR TS=“endocrine“ OR TS=“blindness“ OR TS= “vision loss” OR TS=“trachoma“ OR TS=“cataract*“ OR TS=“glaucoma“ OR TS=“eye disease*” OR TS=“hematologic“ OR TS=“lymphatic“ OR TS=“anemia*“ OR TS=“hemoglobinopath*“ OR TS=“sickle cell” OR TS=“thalassemia*“ OR TS=“G6PD“ OR TS=“GPD“ OR TS=“immun*“ OR TS=“autoimmun*“ OR TS=“lupus“ OR TS=“infectio*“ OR TS=“communicable“ OR TS=”tropical disease*” OR TS=“enteric“ OR TS=“parasitic“ OR TS=“vector borne” OR TS=“viral“ OR TS=“diarrhea*“ OR TS=“coronavirus“ OR TS=“COVID-19“ OR TS=“SARS-CoV-2“ OR TS=“dengue“ OR TS=“nematod*“ OR TS=“loaiasis“ OR TS=“malaria“ OR TS=“influenza“ OR TS=“HIV “OR TS=“AIDS“ OR TS= “acquired immunodeficiency syndrome” OR TS=“tuberculosis“ OR TS=“helicobacter“ OR TS=“H pylori” OR TS=“sexually transmitted infection*” OR TS=“arthritis“ OR TS=“osteoarthritis “OR TS=“rheumat*“ OR TS=”bone disease*” OR TS=”joint disease*” OR TS=“musculoskeletal“ OR TS=“spinal*“ OR TS=“osteoporosis“ OR TS=“gout“ OR TS=“cancer“ OR TS=“cancers“ OR TS=“malignanc*“ OR TS=“neoplas*“ OR TS=“tumor“ OR TS=“tumors“ OR TS=“tumour“ OR TS=“tumours“ OR TS=“carcinoma*“ OR TS=“leukemia*“ OR TS=“lymphoma*“ OR TS=“melanoma*“ OR TS= “nervous system” OR TS=“neurologic“ OR TS=“neurodegenerative“ OR TS=“neuromuscular“ OR TS=“migraine“ OR TS=“meningitis“ OR TS=“dementia*“ OR TS=“alzheimer*“ OR TS= “intellectual disabilit*” OR TS=“nutrition*“ OR TS=“malnutrition*“ OR TS=“deficienc*“ OR TS=“vitamin*“ OR TS=“metaboli*“ OR TS=“hyperlipidemia“ OR TS=“hypercholesterolemia“ OR TS=“dislipidemia*“ OR TS=“lipid*“ OR TS=“disabilit*“ OR TS=“occupational*“ OR TS=“work-related” OR TS=“Environmental Exposure” OR TS=“Otitis“ OR TS= “hearing loss” OR TS= “hearing impairment” OR TS=“mastoiditis“ OR TS=”otorhinolaryngologic” OR TS=“Headache“ OR TS=“backache“ OR TS=“pain” OR TS=“heartburn“ OR TS=“underweight“ OR TS=“overweight“ OR TS=“obesity“ OR TS=“body mass index” OR TS=“chronic obstructive pulmonary disease” OR TS=“asthma“ OR TS=“respirat*“ OR TS=“lung“ OR TS=“pulmonary“ OR TS=“bronchitis“ OR TS=“pneumonia“ OR TS=“skin“ OR TS=“dermatolog*“ OR TS=“dermatitis“ OR TS=“urinary“ OR TS=“infertility“ OR TS=“kidney“ OR TS=“renal“ OR TS=“nephr*“ OR TS=“genital*“ OR TS=“urogenital“ OR TS=“testicular“ OR TS=“urogenital“ OR TS=“Testosterone“ OR TS=“hypogonadism“ OR TS=“gynecol*“ OR TS=“uterine“ OR TS=“ovarian“ OR TS=“cervical“ OR TS=“estrogen“ OR TS=“progestin“ OR TS=“menopaus*“ OR TS=“postmenopaus*“ OR TS=“pap smear” OR TS=“mammogra*“ OR TS=“colonoscopy“ OR TS= “fecal occult blood” OR TS=“Cholesterol“ OR TS=“prostate screening” OR TS=“alcohol screening” OR TS=“Immuniz*“ OR TS=“Vaccin*” OR TS=“poisoning“ OR TS=“substance use” OR TS=“Illicit drug*” OR TS=“drug use” OR TS=“alcohol use” OR TS=“alcohol abuse” OR TS=“nicotine“ OR TS=“vaping“ OR TS=“e-cigarette” OR TS=“tobacco use” OR TS=“Palliative“ OR TS=“end of life” OR TS=“Terminal Care”)

TS=”access*” OR TS=”accept*” OR TS=”attitude*” OR TS=”aware*” OR TS=”barrier*” OR TS=”boundar*” OR TS=”challenge*” OR TS=”deliver*” OR TS=”disadvantage*” OR TS=”dissatisf*” OR TS=”disparit*” OR TS=”enable*” OR TS=”gap” OR TS=”engage*” OR TS=”experience*” OR TS=”facilitat*” OR TS="gaps” OR TS=”help seeking” OR TS=”helpseeking” OR TS=”health policy” OR TS=”inequality” OR TS=”inequalities” OR TS=”equity” OR TS=”inequity” OR TS=”inequities” OR TS=”knowledge” OR TS=”motivat*” OR TS=”obstacle*” OR TS=”perceiv*” OR TS=”perception*” OR TS=”perspective*” OR TS=”promotion” OR TS=”satisf*” OR TS=”trust” OR TS=”understand*” OR TS=”uptake” OR TS=”usage” OR TS=”utilis*” OR TS=”utiliz*” OR TS=“electronic access” OR TS=“medical record” OR TS=”electronic health” OR TS=”insurance” OR TS=”insured ”OR TS=”uninsur*” OR TS=”underinsur*” OR TS=”interpret*” OR TS=”education” OR TS=”communication” OR TS=”literacy” OR TS=”social isolation” OR TS=”social marginalization” OR TS=”social environment” OR TS=”social conditions” OR TS=”determinant*” OR TS=”psychosocial” OR TS=”sociodemographic” OR TS=”discrimination” OR TS=”caregiv*” OR TS=”caregiver*” OR TS=”incarcerat*” OR TS=”socioeconomic” OR TS=”income” OR TS=”employment” OR TS=”workplace” OR TS=”work-related” OR TS=”unemploy*” OR TS=”impoverished*” OR TS=”poverty*” OR TS=”cost” OR TS=”costs” OR TS=”neighborhood* ”OR TS=”liveable” OR TS=”livable” OR TS=“built environment" OR TS=”housing” OR TS=”foreclosure” OR TS=”eviction” OR TS=”mortgage” OR TS=”physical activity" OR TS=”exercise” OR TS=”sedentary behavior" OR TS=”walkability” OR TS=”walkable” OR TS=”transportation” OR TS=”food” OR TS=”crime” OR TS=”violence” OR TS=”Acculturation” OR TS=”Social Isolation” OR TS=”Marginalization”

TS="Treatment Adherence" OR TS="non-compliance" OR TS="noncompliance" OR TS="compliance" OR TS="non-adherence" OR TS="nonadherence" OR TS="adherence" OR TS="refusal" OR TS="no show" OR TS="non-attendance" OR TS="mobile health" OR TS="mhealth" OR TS="m-health" OR TS="ehealth" OR TS=" e-health" OR TS="app" OR TS="apps" OR TS="application" OR TS="applications" OR TS="software" OR TS="digital health" OR TS="patient portal" OR TS="patient portals" OR TS="Mobile Applications" OR TS="Wearable Electronic" OR TS="telehealth" OR TS="telemedicine" OR TS="remote monitoring" OR TS="tele-medicine" OR TS="telehealth" OR TS="tele‐health" OR TS="telecare" OR TS="tele-care" OR TS="tele‐homecare" OR TS="telehomecare" OR TS="telemanag*" OR TS="tele‐manag*" OR TS="telemonitor*" OR TS="tele‐monitor*" OR TS="teleconsultation*" OR TS="tele-consultation*" OR TS="telenurs*" OR TS="tele‐nurs*" OR TS="remote consultation" OR TS="virtual clinic" OR TS="virtual clinics" OR TS="virtual visit" OR TS="virtual visits" OR TS="virtual care" OR TS="video consult" OR TS=videoconsult*

**CINAHL via EBSCOHost**

(refugee* OR asylee* OR "asylum seeker" OR "asylum seekers" OR "asylum seeking" OR (MH "Refugees")

Search mode: Proximity Search, Turn off Apply related words, Also search within the full text of the articles, and Apply Equivalent Subjects
