# Supplementary figures and images for: Interventions to Improve Health Among Refugees in the United States: A Systematic Review
Source: J Community Health. 2024 Sep 6;50(1):130–51. doi: 10.1007/s10900-024-01400-2 (PMC11805879; doi:10.1007/s10900-024-01400-2)

*Supplement 2 – Quality Appraisal of Included Studies*
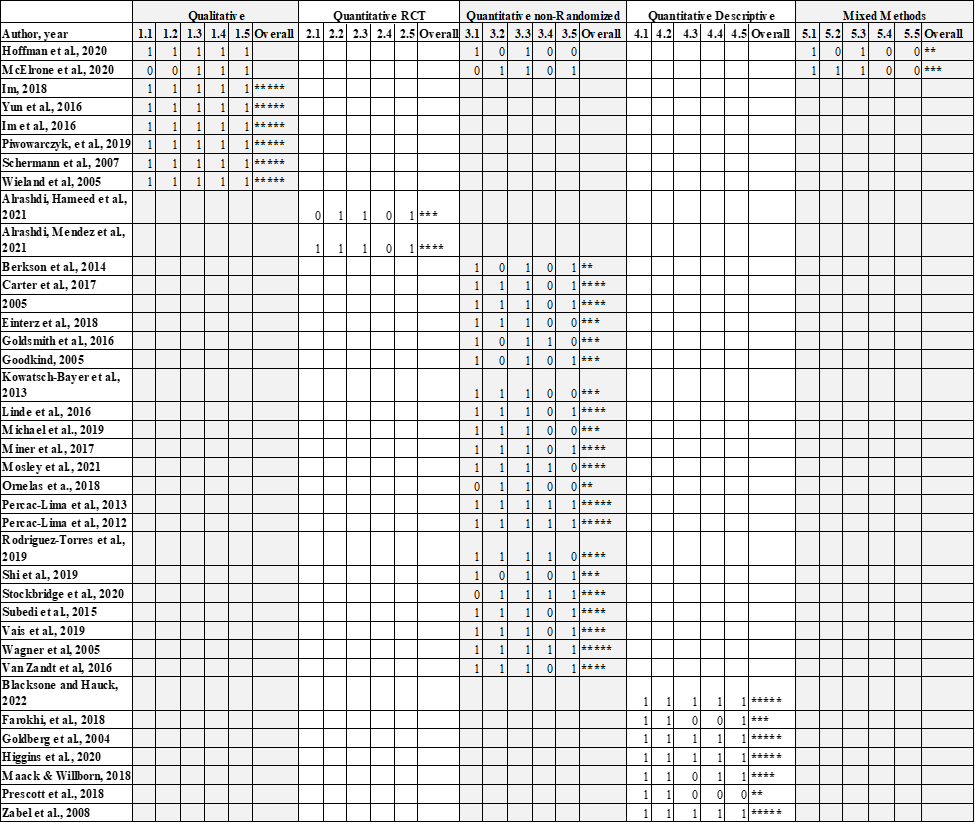

Supplement: Supplementary file 2 — Supplementary Material 2 [file 10900_2024_1400_MOESM2_ESM.docx]
